# Proteomic profiling of single extracellular vesicles reveals colocalization of SARS-CoV-2 with a CD81/integrin-rich EV subpopulation in sputum from COVID-19 severe patients

**DOI:** 10.3389/fimmu.2023.1052141

**Published:** 2023-05-12

**Authors:** Ruiting Sun, Yanling Cai, Yumin Zhou, Ge Bai, Airu Zhu, Panyue Kong, Jing Sun, Yimin Li, Yuefei Liu, Wenting Liao, Jiye Liu, Nan Cui, Jinsheng Xiang, Bing Li, Jincun Zhao, Di Wu, Pixin Ran

**Affiliations:** ^1^ National Center for Respiratory Medicine, State Key Laboratory of Respiratory Disease, National Clinical Researcher Center for Respiratory Diseases, Guangzhou Institute of Respiratory Health, The First Affiliated Hospital of Guangzhou Medical University, Guangzhou, Guangdong, China; ^2^ Shenzhen Second People’s Hospital, Postdoctoral Workstation of Zhongshan School of Medicine, Sun Yat-Sen University, Shenzhen, Guangdong, China; ^3^ R&D Department, Shenzhen Secretech Co. Ltd, Shenzhen, Guangdong, China; ^4^ R&D Department, Vesicode AB, Solna, Sweden

**Keywords:** COVID-19, SARS-CoV-2, single extracellular vesicles, inflammatory response, extracellular vesicles subpopulation

## Abstract

**Background:**

The global outbreak of COVID-19, and the limited availability of clinical treatments, forced researchers around the world to search for the pathogenesis and potential treatments. Understanding the pathogenesis of SARS-CoV-2 is crucial to respond better to the current coronavirus disease 2019 (COVID-19) pandemic.

**Methods:**

We collected sputum samples from 20 COVID-19 patients and healthy controls. Transmission electron microscopy was used to observe the morphology of SARS-CoV-2. Extracellular vesicles (EVs) were isolated from sputum and the supernatant of VeroE6 cells, and were characterized by transmission electron microscopy, nanoparticle tracking analysis and Western-Blotting. Furthermore, a proximity barcoding assay was used to investigate immune-related proteins in single EV, and the relationship between EVs and SARS-CoV-2.

**Result:**

Transmission electron microscopy images of SARS-COV-2 virus reveal EV-like vesicles around the virion, and western blot analysis of EVs extracted from the supernatant of SARS-COV-2-infected VeroE6 cells showed that they expressed SARS-COV-2 protein. These EVs have the infectivity of SARS-COV-2, and the addition can cause the infection and damage of normal VeroE6 cells. In addition, EVs derived from the sputum of patients infected with SARS-COV-2 expressed high levels of IL6 and TGF-β, which correlated strongly with expression of the SARS-CoV-2 N protein. Among 40 EV subpopulations identified, 18 differed significantly between patients and controls. The EV subpopulation regulated by CD81 was the most likely to correlate with changes in the pulmonary microenvironment after SARS-CoV-2 infection. Single extracellular vesicles in the sputum of COVID-19 patients harbor infection-mediated alterations in host and virus-derived proteins.

**Conclusions:**

These results demonstrate that EVs derived from the sputum of patients participate in virus infection and immune responses. This study provides evidence of an association between EVs and SARS-CoV-2, providing insight into the possible pathogenesis of SARS-CoV-2 infection and the possibility of developing nanoparticle-based antiviral drugs.

## Graphical Abstract

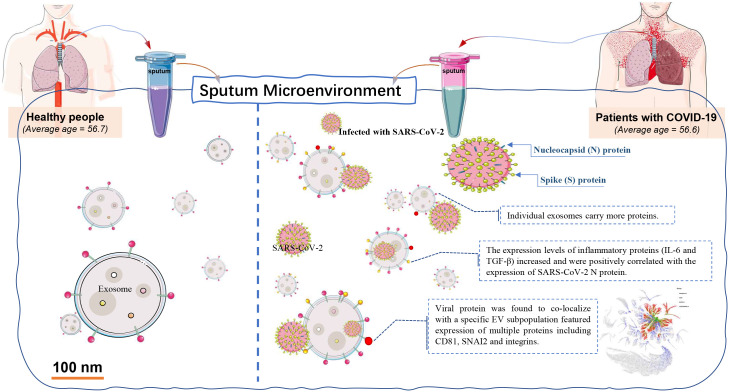

## Highlights

- Extracellular vesicles are considered mediators of cell-cell communication as well as intercellular ferries of a diverse cargo of proteins, lipids, and nucleic acids. However, little is known about the role of EVs during SARS-CoV-2 infection and in subsequent immune responses.
- Our study suggests that sputum EVs from COVID-19 patients carried SARS-CoV-2 N protein;
- EVs are involved in the immune response of SARS-CoV-2;
- Viral protein colocalized with a specific EV subpopulation expressing multiple proteins;
- Single-EV harbor infection-mediated alterations in proteins.

## Introduction

As of April 28, 2022, there were more than 179.27 million confirmed cases of coronavirus disease 2019 (COVID-19) worldwide, with more than 6,235,642 deaths reported to Worldometers. The worldwide spread of COVID-19 and the scarcity of clinical remedies compelled researchers from various countries to explore potential treatments (1). Patients with COVID-19 who experience excessive inflammation and immune responses may have severe clinical progression. A considerable proportion of individuals suffer from severe pneumonia, and some even develop acute respiratory distress syndrome (ARDS) (2), which is believed to be triggered by cytokine storms (IL-6, IL-1, and TNF-α). Accordingly, treatments such as corticosteroids, which control inflammatory cytokine signaling, are used to reduce the mortality of patients with COVID-19 (3–5). Extracellular vesicles (EVs), especially exosomes, have emerged as key mediators of various physiopathological processes related to virus infection and are actively involved in mediating responses, both beneficial and detrimental, to virus-induced injury (6) and inflammation (7).

Exosomes, which are functional vehicles secreted by various types of cells, have a diameter of 30–130 nm and carry a complex cargo of proteins, lipids, and nucleic acids. It has been proven that the hepatitis A virus can hijack exosome membranes and transport virus pathogenesis-related proteins (8), genomes, and even virus particles to all parts of the body using the characteristics of the free shuttle, which is the ability of exosomes to shuttle between host and target cells (9). Blocking vesicle release from hepatitis C virus (HCV)-positive cells increases intracellular dsRNA levels and activates toll-like receptor 3, thereby inhibiting HCV replication (10). Therefore, we speculated that EVs might also be involved in trafficking severe acute respiratory syndrome coronavirus-2 (SARS-CoV-2). Indeed, EV-like vesicles may mediate the spread of SARS-CoV-2 throughout the lungs. However, little is known about the role of EVs during SARS-CoV-2 infection and in subsequent immune responses. Furthermore, the EV subsets in sputum samples, and subsequent changes in their proteomic features during SARS-CoV-2 infection, remain poorly understood. Therefore, it is of great interest to examine the relationship between EVs and SARS-CoV-2. In particular, identification of proteins transmitted by EVs would help identify potential drug targets, and/or enable reuse of existing drugs depending on specific protein expression profiles.

The aim of this study was to use a library of DNA-tagged antibodies to identify proteins co-expressed by EVs and SARS-CoV-2. EVs obtained from healthy donors and COVID-19 patients were examined. Meanwhile, the proportion of each subpopulation was quantified, and their proteomic fingerprint was profiled. Then, co-expression of the SARS-CoV-2 N protein with other EV proteins was selected from the protein combination data set and analyzed to predict virus-EV association. The data may increase our knowledge of the EV subsets involved in the pathogenesis of the COVID-19, which would facilitate the design of therapeutic strategies to fight SARS-CoV-2 infection.

## Materials and methods

### Selection of patients and healthy subjects

**Figure 1:**
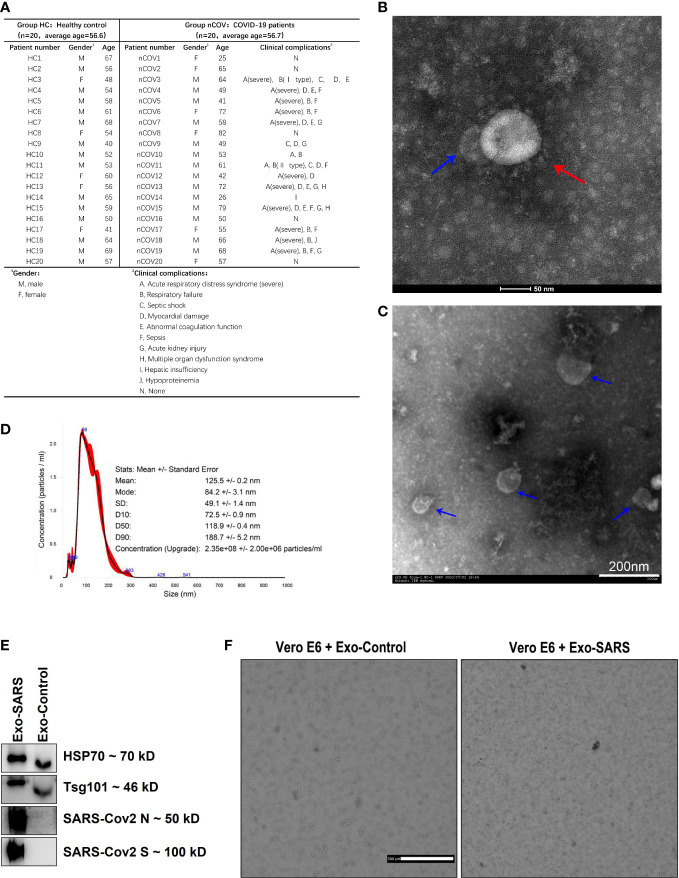
Demographic information and characterization of EVs from the sputum of COVID-19 patients and controls. Sputum was collected from patients in the ICU. Twelve of the 20 patients required ventilation as they were diagnosed with acute respiratory distress syndrome. **(A)** Donor information and the clinical complications of COVID-19 infected patients. **(B, C)** Morphology of SARS-CoV-2 particles (red arrows) and EVs (blue arrows), as assessed by Transmission Electron Microscopy. **(D)** Nanoparticle tracking analysis of particles in sputum samples from COVID-19 patients (50 μl of EVs was extracted from 100 μl of sputum and analyzed after being diluted 200-fold). The inset shows the size distribution (black lines) of EVs (no magnet was used). The error bars (in red) indicate the standard error of the mean. **(E)** Exosomes from supernatants of normal and SARS-CoV-2 infected VeroE6 cells (labeled as Exo-Control and Exo-SARS, respectively) were examined by western blotting. **(F)** The exosomes mentioned in E were added to normal VeroE6 cells. After 48 hours, the cells that were exposed to Exo-Control (left image) proliferated normally, while cells exposed to Exo-SARS (right image) died.

The present study was approved by the Ethics Committee of the First Affiliated Hospital of Guangzhou Medical Univers. The HC group included 20 healthy donors without symptoms such as cough, allergy, and respiratory tract discomfort (3). The nCOV group included 20 patients with RT-PCR-confirmed infection by SARS-CoV-2 (Daan Gene Co., Ltd.; Guangzhou, China) (11). The control population was selected based on the average age of the randomized patients, and the two groups ended up being equally old on average. The clinic-pathological conditions of the patients are shown in **Figure 1A**.

### Detection of SARS-CoV-2

SARS-CoV-2 was detected by real-time RT-PCR (11). Nucleic acid was extracted from sputum samples using a Viral RNA extraction kit (Daan Gene Co., Ltd.; Guangzhou, China). RNA extraction from sputum and blood was performed using a total RNA extraction kit (Sangon Biotech; Shanghai, China). The real-time PCR assay targeting the SARS-CoV-2 RdRp and N gene regions was provided by Daan Gene Co., Ltd.

### Sputum sample collection and pretreatment

Sputum was collected from patients during a pulmonary exacerbation (all were in a stable condition) by inhalation of hypertonic (NaCl 5%) or isotonic (NaCl 0.9%) saline. Similarly, sputum was collected from normal healthy people (HC group). Sputum samples were observed under a microscope to ensure that they met the inclusion criterion. All operations were performed in a Biosafety laboratory (P3) (2).

### Virus isolation and transmission electron microscopy

Vero E6 cells were used for virus isolation. A quantitative reverse transcription PCR (qRT-PCR)-positive sputum swab specimen was saved in viral transport medium (DMEM containing 1% bovine serum albumin, 15 µg/mL amphotericin, 100 units/mL penicillin G, and 100 μg/mL streptomycin). Before virus isolation, the sample was passed through a 0.45 μm strainer and diluted 1:10 with DMEM containing 2% FBS and antimicrobial drugs. Cells were infected at 37°C for 1h. The inoculum was removed and replaced with a fresh culture medium. Cytopathic effects (CPE) were observed in Vero E6 cells infected with SARS-CoV-2 isolates after 72h. No CPE were observed in mock-infected cells. The morphology of SARS-CoV-2 was visualized by transmission electron microscopy (H-7650, Hitachi, Japan) (11).

### Characterization of EVs in the sputum sample and cell culture supernatant

EVs were isolated using a standard ultracentrifugation protocol after initial extraction using the EV extraction kit (ExoQuick-TC, SBI, USA). The concentration and size distribution of EVs were investigated using a NTA system (Nanosight NS300, Malvern Panalytical Ltd., UK) (12). EV morphology was examined under transmission electron microscope. Expression of exosome surface marker proteins was examined by western blotting.

### EV capture, fixation, and permeabilization

The streptavidin-coated PCR plates (PCR0STF-SA5/100, Biomat, Italy) were incubated with biotinylated cholera toxin subunit B (2.5 μg/ml; C34779, Thermo-Fisher Scientific, USA) in PBS (C10010500BT, Gibco, USA) at room temperature for 2 h. Then, the plate wells were rinsed three times with PBST washing buffer (0.05% Tween-20 (003005, Thermo-Fisher Scientific, USA) in PBS). Afterward, 20 μl of sputum/PBS was added to the wells of the plate. Wells were rinsed with PBST after incubation at room temperature for 2 h. The fixation step was performed by adding 20 μl of 4% paraformaldehyde/PBS (BL539A, Biosharp, China) to each well. Thereafter, 0.2% Triton-X (T8787, Sigma-Aldrich, USA) in PBS was added to allow detection of proteins within EVs. Finally, the wells of the plate were rinsed three times with PBST.

### Single EV proteomics analysis using the Proximity Barcoding Assay template

The experimental method for the PBA was performed as described previously (13). Here, a CTB-coated surface was reacted with ganglioside GM1 (which is enriched in membrane lipid rafts) to capture EVs. For EV proteomic analysis, 100 antibodies were conjugated with DNA oligonucleotides comprising an 8-nucleotide (nt) protein tag, an 8-nucleotide (nt) molecule tag, and universal sequences as adapters. The proteins under investigation included typical EV biomarkers, biomarkers related to lung diseases, and a panel of cell adhesion molecules. The PBA tests were designed according to the protocols published by Vesicode AB (Solna, Sweden) and were performed by Secretech (Shenzhen, China).

### Data processing

After the DNA sequencing, raw data were obtained in bcl file format. After running the bcl2fastq program (Illumina, USA), fastq files for each sample were generated. Using fastx_toolkit, low-quality reads (Phred quality score Q <20) were removed before further analysis. The clean data files for each sample constituted DNA reads of 75 bp, and the EV tag, protein tag, and molecule tag were extracted. The molecule tags were used to deduplicate the amplified sequences for library construction, and the unique reads were used for the subsequent assays. The protein tags were translated to the protein name by matching to the antibody-DNA tag conjugation list (**Supplementary Information Table 1**). The EV-protein matrix contained columns showing protein expression and rows for single EVs in each sample, as indicated by the detected EV tags (**Supplementary Information Table 2**).

The EV-associated protein expression levels were obtained by summating the quantity of a certain protein detected on all EVs. The data was normalized using the count per million (CPM) reads method, accounting for the library size, and the trimmed mean (TMM) method, accounting for composition bias. The protein combinations are the information of the protein co-expression on the same EV. An unsupervised machine learning algorithm, FlowSOM, was applied to cluster EVs, according to the proteomic features of EVs. The number of clusters was determined according to the consensus matrix, in which the lowest number of clusters for optimal separability was selected. The proteomic similarity of EVs was observed in the T-distributed Stochastic Neighbor Embedding (t-SNE) plot. The proportion of each subpopulation was quantified. The proteomic fingerprints for each subpopulation were profiled.

The protein combinations were summarized in the format of the EV tag-(p1, p2, p3…). The quantity of each possible pair of co-expressed protein was obtained as the protein combination dataset, and used as input variables for the abundance and differential analysis. The differential analysis between the nCOV and HC groups was performed and visualized. The co-expression of SARS-CoV-2 protein SARS-CoV-2 N with EV proteins was selected from the protein combination dataset and analyzed to predict virus-EV association.

### Quantification and statistical analysis

For PBA data analysis, edger package was applied to identify differentially expressed proteins and protein combinations (13). Differences in mean values between groups were analyzed by ANOVA. All analyses were performed in GraphPad Prism 7, and results are expressed as the mean ± standard error of the mean (SEM). P values <0.05 were considered statistically significant. *P ≤ 0.05, **P ≤ 0.005, ***P ≤ 0.001, and ****P ≤ 0.0001.

## Results

### Demographic information of COVID-19 patients and characterization of EVs harboring SARS-CoV-2

Twenty patients with severe COVID-19 (nCOV group) and 20 healthy controls (HC group) were enrolled; most were aged over 50 years, with an average age of 56.6 and 56.7 years, respectively. Sputum was collected from COVID-19 patients when they were still in the ICU. Twelve of the 20 patients required ventilation as they were diagnosed with ARDS. The patient information is provided in **Figure 1A**.

The SARS-CoV-2 virus was isolated successfully from the sputum supernatant of COVID-19 patients (**Figure 1B**, red arrow). Surprisingly, EV-like vesicles were found close to the virions (**Figure 1B**, blue arrow). Next, EVs were isolated from sputum by differential ultracentrifugation. Electron microscopy revealed that the EVs were cup-shaped and had a lipid bilayer membrane vesicle structure (**Figure 1C**). To further characterize the nature of the released vesicles, particle tracking was performed using a NanoSight instrument. Nanoparticle tracking analysis (NTA) enabled us to obtain particle size distribution profiles and to perform concentration measurements. As shown in **Figure 1D**, the primary peak was observed at approximately 86 nm, consistent with the size of most EVs (30–200 nm). The size distribution was quite monodispersed. The concentration of particles in sputum samples from COVID-19 patients was 2.35×10^8^ ± 2×10^6^/ml.

In addition, western blot analysis of EVs isolated from the supernatant of normal and SARS-CoV-2-infected VeroE6 cells (labeled as Exo-Control and Exo-SARS, respectively) expressed exosome surface markers HSP70 and Tsg101 (**Figure 1E**). More importantly, exosomes extracted from the latter expressed SARS-CoV-2 N and S protein (**Figure 1E**). Furthermore, when we added Exo-SARS to normal VeroE6 cells, they died after 48 h in a manner similar to that of virus-infected cells (**Figure 1F**). This strongly suggests that exosomes are involved in virus transmission.

### Patients with COVID-19 secrete more proteins in individual EVs, and EVs participate in the immune response

**Figure 2:**
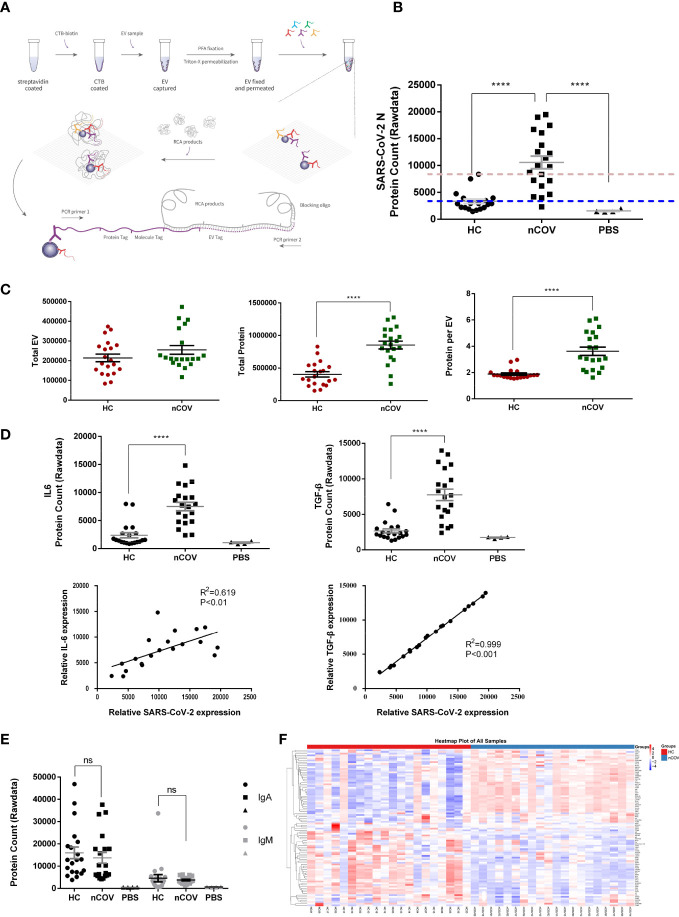
Patients with COVID-19 secrete more protein in individual EVs, and EVs play a role in the immune response to SARS-CoV-2. (nCOV, n = 20; HC, n = 20; and PBS, n = 4) **(A)** Proximity Barcoding Assay (PBA) template for analysis of the protein profile at the single EV level. A lipid membrane binding layer (streptavidin-biotin-CTB coated onto the wall of a 0.2 ml well) was used to capture EVs from each sample. After library construction and sequencing, original data were obtained in fastQ file format. After quality control and tag extraction, the file containing individual EVs and the proteins detected in each sample were summarized. **(B)** Quantification of EVs and proteins detected in the PBA, and the percentage of each protein expressed by each EV from COVID-19 patients (nCOV group) and healthy controls (HC group). When the mean signal value for the control group was set as the baseline (the blue dotted line), 19 of 20 individuals harbored the SARS-CoV-2 N protein in EVs. However, when the maximum signal value for the control group was set as the baseline (the red dotted line), 12 of 20 individuals harbored the SARS-CoV-2 N protein in EVs. **(C)** SARS-CoV-2 N protein signals were detected in EVs obtained from nCOV, HC, and PBS controls. **(D)** Quantification of IL6 and TGF-β, and correlation with SARS-CoV-2 N protein in the nCOV and HC groups. **(E)** Quantification of IgA and IgM proteins. ****P < 0.0001 (Student’s t-test). **(F)** Total protein expression in sputum EV samples from the nCOV and HC groups. The heatmap shows the proteomic profile of the samples. ns, not significant.

Coexpressed EV and virus proteins were identified using PBA. EVs were captured on a CTB-coated surface that interacts with ganglioside GM1 in membrane lipid rafts. The scheme of the workflow is illustrated in **Figure 2A**. The antibody-conjugated oligonucleotides were brought into the proximity of the same EV *via* protein-antibody interactions, thereby barcoding the EV (13).

In each 5 μl sputum sample, the mean number of EVs detected by PBA tended to increase after SARS-CoV-2 infection (**Figure 2C**, left panel), and the number of proteins detected in EVs from the nCOV group was twice that in those from the HC group (**Figure 2C**, middle panel). With respect to the proteins under investigation, sputum EVs from the nCOV group had higher amounts (3.6 proteins/EV) than the HC group (1.9 proteins/EV) (**Figure 2C**, right panel). As mentioned in the Methods, 20 nCOV, 20 HC, and four PBS controls were examined. As shown in **Figure 2B**, SARS-CoV-2 N protein signals were detected in EVs obtained from COVID-19 patients (**Figure 2B**). The protein signal in some individuals from the control group was slightly higher than that of PBS, which is an acceptable systematic error due to nonspecific binding of antibodies.

Consistent with previous reports on expression of cytokines in serum (14), we found that expression of IL6 and TGF-β was higher in EVs from COVID-19 patients. Furthermore, this increase showed a strong correlation with SARS-CoV-2 N protein expression (**Figure 2D**). We also found that expression of other proteins increased significantly after SARS-CoV-2 infection; these included the T cell activation marker CD26, human leukocyte antigen HLA-A, and adhesion molecule MAdCAM-1(**Figure S1**), which is overexpressed in inflamed mucosal tissue. These results suggest that EVs play a role in the immune response to COVID-19. However, although IgA levels in sputum EVs from COVID patients tended to be higher than those of IgM (consistent with the findings in serum (15)), there was no significant difference in the total expression of IgA and IgM in sputum EVs of healthy controls and patients (**Figure 2E**). After trimmed mean method (TMM) normalization, a protein expression heatmap (**Figure 2F**) revealed that nCOV patients showed a general shift in the EV proteomic profile compared with HC samples, although there were exceptions. Differentially expressed proteins were analyzed in a volcano plot after normalizing the TMM protein expression data, followed by generation of a dot plot (**Figure S2**).

### The EV subpopulation atlas, and changes in patients with COVID-19

**Figure 3:**
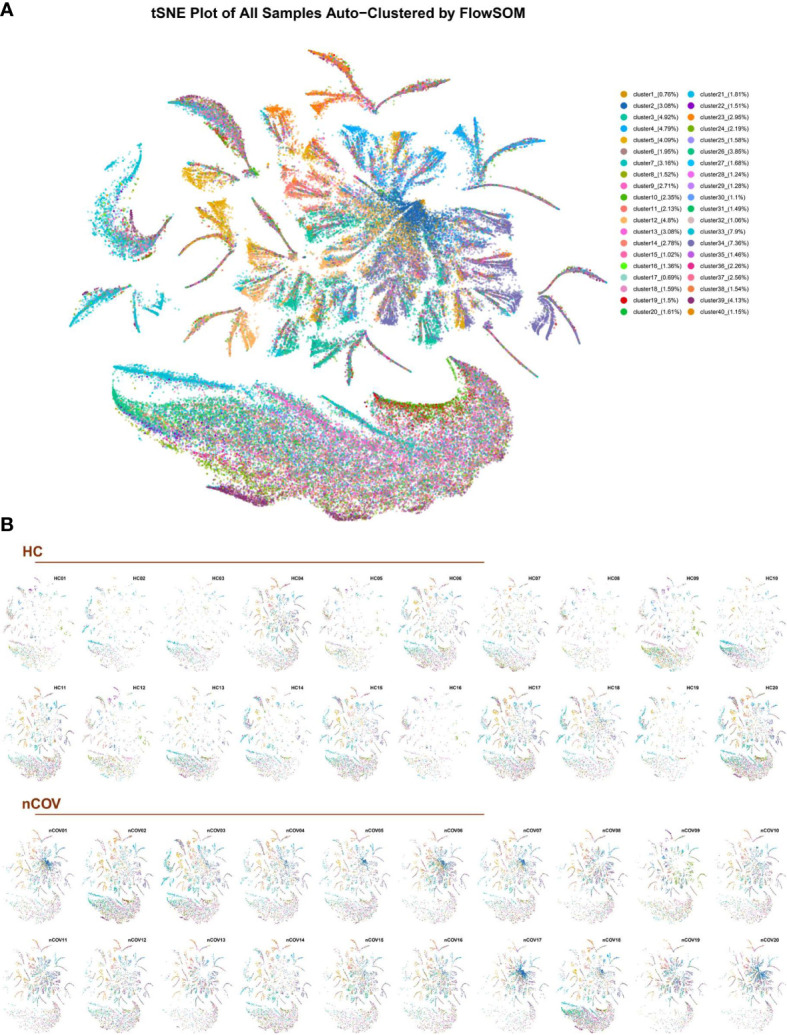
The EV subpopulations in 40 different samples, as determined by an unsupervised machine learning process (FlowSOM). **(A)** Forty subpopulations were displayed on a t-distributed stochastic neighbor embedding (tSNE) plot. The tSNE representation of EVs from all analyzed samples (n = 40) was colored by manually annotating the EV type. **(B)** tSNE plots for each sample obtained from healthy individuals (HC, n = 20) and COVID-19 infected patients (nCoV, n = 20). tSNE was conducted for each cluster.

The algorithm FlowSOM was used to analyze the behavior of all markers expressed by all individual EVs; then, clusters of EVs were generated using a self-organizing map. The clusters, which represent EV subpopulations, were determined according to the proteomic fingerprint of each EV. We detected 9,377,119 EVs, with an average of 234,428 EVs per sample (**Figure 2C**). Dimensionality reduction indicated substantial phenotypic similarities and differences between COVID-19 patients and controls. The t-distributed stochastic neighbor embedding (tSNE) plot for each sample (**Figure 3A**) identified 40 clusters. **Figure 3B** shows the 40 clusters as different colors.

**Figure 4:**
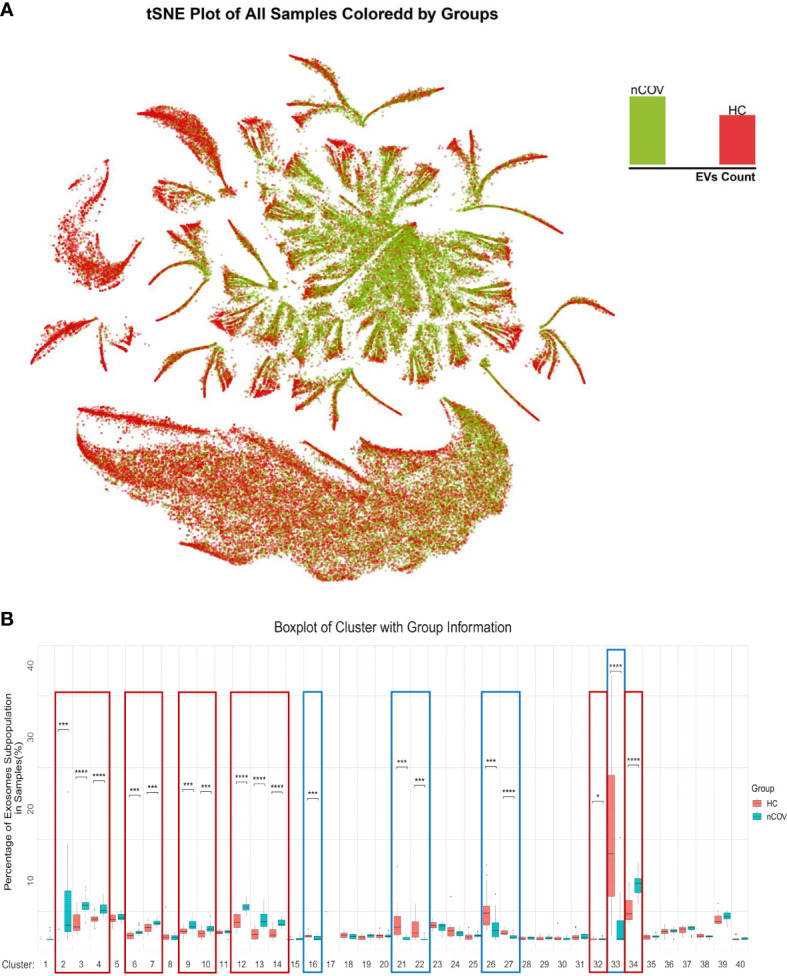
EV subpopulation atlas and changes specific to patients with COVID-19. **(A)** Similarities and differences in EV proteomics between sputum samples from COVID-19 patients and HC groups; all EVs in the nCoV group are green, and all EVs in the HC group are red. **(B)** Quantification of the EV subpopulations in the nCOV and HC groups. Compared with the HC group, the nCOV group had a significantly high ratio of EV subpopulations in clusters 2, 3, 4, 6, 7, 9, 10, 12, 13, 14, 32, and 34, but a decreased ratio of subpopulations in clusters 16, 21, 22, 26, 27, and 33, than HCs (*P < 0.05; ***P < 0.001; and ****P < 0.0001; Student’s t-test).

Next, we used a modeling approach to detect characteristics that distinguish healthy individuals from infected individuals. **Figure 4A** shows similarities and differences in EV proteomics between sputum samples from the nCOV and HC groups. Color-coding the subpopulations of EVs in this way enables them to be distinguished more easily (**Figure 4A**). The tSNE plot revealed proteomic similarities between the EVs. Next, we calculated the proportion of each subpopulation. Among the 40 subpopulations, 18 clusters showed significant differences. These were cluster 2, 3, 4, 6, 7, 9, 10, 12, 13, 14, 32 and 34, which had a significantly elevated ratio of EV subpopulations in the nCOV group, while the ratio for the subpopulation decreased in other clusters (**Figures 4B**, **S3**).

**Figure 5:**
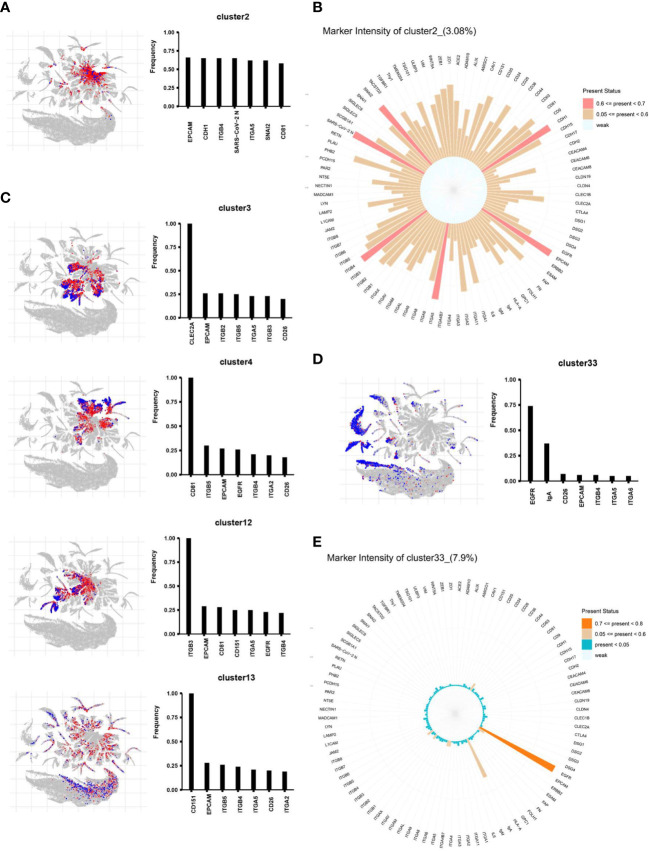
The differential distribution of EVs obtained from the nCOV and HC groups. The location of each differentially expressed EV subpopulation within the total EV population is shown, along with the top seven expressed proteins. **(A, B)** Cluster 2 comprises 4.92% of all EVs in the nCOV group, but only 0.55% in the HC group. The EVs in cluster 2 show high expression of SARS-CoV-2 N protein, along with the EV biomarker CD81 and cell adhesion molecules EpCAM, CDH1, ITGB4, ITGA5, and SNAI2. **(C–E)** The proteomic profile of clusters 3, 4, 13, and 33. Cluster 33 in the HC group shows high expression of EGFR and IgA (14.12% of EVs in the HC group *vs*. 3.37% of EVs in the nCOV group).

Among these, clusters 2, 3, 4, 12, 13, 34, and 33 accounted for most of the differences. We then analyzed these seven clusters in more detail by profiling their proteomic fingerprints. For each differentially expressed EV subpopulation, its location within the total EV population, and the top seven proteins expressed by these EVs, are shown in **Figure 5**. First, we focused on cluster 2, which comprised 4.92% of all EVs in the nCOV group but only 0.55% in the HC group (**Figures 5A**, **S3**). The EVs in cluster 2 contained a large amount of SARS-CoV-2 N protein. This suggests that these EVs make direct contact with the SARS-CoV-2 virus or are secreted by the cells in which the virus replicates. These EVs expressed high levels of the exosome biomarker CD81, as well as cell adhesion molecules. **Figure 5B** shows the proteomic characteristics of cluster 2 (100 proteins). As shown in **Figure 4B**, Clusters 3, 4, 12, 13, and 34 represent EVs that increased in the nCOV group, and showed expression of CLEC2A, CD81, ITGB3, CD151, and ITGB2, respectively (**Figures 5C**, **S3**). By contrast, the amount of cluster 33 in the nCOV group was lower than that in the HC group (3.37% *vs*. 14.12%, respectively). Cluster 33 showed high expression of EGFR and IgA (**Figures 5D, E** **)**.

### EVs regulated by CD81 are more likely to carry SARS-CoV-2 proteins

**Figure 6:**
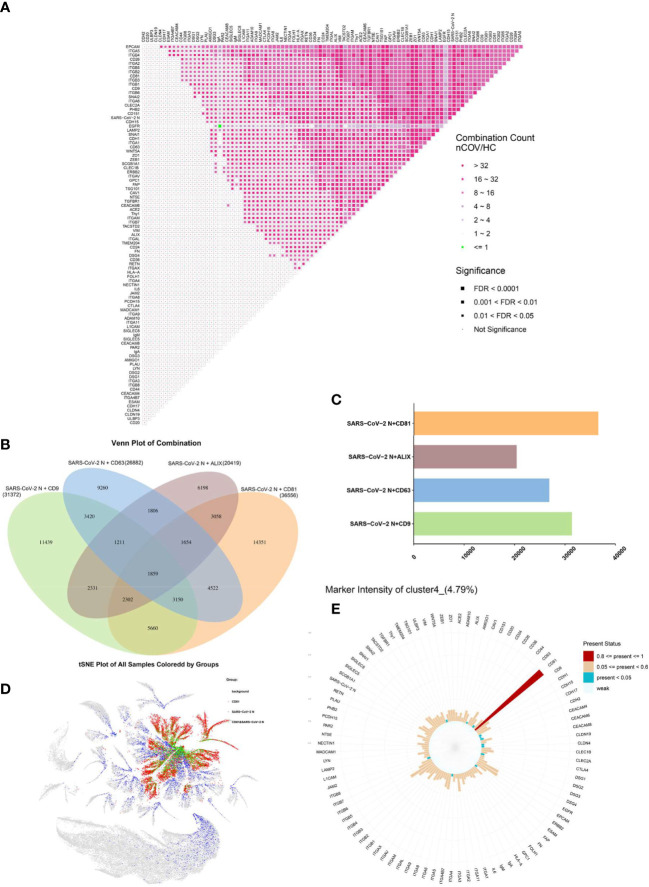
EVs regulated by CD81 are more likely to carry SARS-CoV-2. **(A)** Protein combinations in the matrix. The -fold change in expression is color-coded, and the significance is indicated by the size of the dots. **(B, C)** Colocalization of the SARS-CoV-2 N protein with EV biomarkers. **(B)** Venn diagram analysis of multiple data sets. **(C)** Levels of enriched colocalization. The number of associated query proteins within a term is shown on the right side of each term bar. **(D)** Distribution of CD81 (red) and SARS-CoV-2 N (blue) proteins in all EVs; co-expression is shown in green. **(E)** Proteomic profile of cluster 4.

“Protein combinations” were defined as colocalization of two proteins within the same individual EV; as such, they can be considered as fingerprints of individual EVs. The quantity of each possible pair of co-expressed proteins was obtained as a protein combination data set, which was then used as an input variable for abundance and differential analysis (**Figure 6A**). The protein combinations exhibited a universally increasing trend in the nCOV group; the exceptions were the combinations EGFR and IgA. In addition, co-expression of integrin subgroups increased significantly.

To investigate colocalization of viral protein and EVs, we further analyzed the combination of SARS-CoV-2 N protein with other proteins in individual EVs. Among markers that regulate EVs, we examined co-expression of CD9, CD63, CD81, and Alix with the SARS-CoV-2 N protein (**Figure 6B**) and found that the EVs regulated by CD81 were more likely to bind to the SARS-CoV-2 N protein (**Figure 6C**). In addition, we found that cluster 2 (**Figure 5B**), cluster 4 (**Figure 5C**), cluster 6, cluster 7, cluster 12 and cluster 34 were the EVs that were highly expressed after SARS-CoV-2 infection, while CD81 was highly expressed in them. In particular, the protein matrix of cluster 4 revealed that expression of CD81 was abnormally high (**Figure 6E**). These results suggest that EVs regulated by CD81 are the most likely subpopulations that cause changes to the pulmonary microenvironment after SARS-CoV-2 infection. The distribution of CD81 and SARS-CoV-2 N protein expression in all EVs is shown in **Figure 6D**.

## Discussion

In this study, we isolated and identified EVs from the sputum of COVID-19 patients to investigate EV-mediated inflammatory and immune responses. We found that EV-like vesicles coexisted alongside virions (**Figure 1B**), and that the mean number of EVs increased after SARS-CoV-2 infection (**Figure 2C**). The nucleocapsid protein of SARS-CoV-2 (SARS-CoV-2 N) is an important structural protein that binds to viral RNA, thereby playing an important role in virus packaging and other processes (16). As expected, the SARS-CoV-2 N protein was detected in EVs obtained from patients (**Figure 2B**). The results suggest a possible role of EVs in SARS-CoV-2 transmission. For example, membrane hijacking by SARS-CoV-2 may promote virus spread through exosome-like vesicles; this possibility should be examined in further studies.

The amount of protein encapsulated in EVs obtained from sputum was significantly higher in patients (**Figure 2C**), suggesting that viral infection stimulates EV secretion. Cell and animal models of SARS-CoV-2 infection (17), in addition to serum profiling of COVID-19 patients, consistently show a unique and inappropriate inflammatory response (18). Here, we detected increased expression of IL-6 and TGF-β by EVs obtained from COVID-19 patients (**Figure 2D**), which is in agreement with previous results obtained from peripheral blood (19). Furthermore, expression of IL-6 and TGF-β by EVs correlated with that of SARS-CoV-2 N protein. In addition, we found that most integrins and other adhesion molecules were also upregulated (**Figure S1**), which may also affect the interaction between immune cells and the local microenvironment (20, 21). All of these data indicate that EVs play a role in the immune response to COVID-19 infection. Secretory IgA plays an important role in protection and homeostatic regulation of the respiratory mucosal epithelium, a process referred to as “immune exclusion” (15). However, we found no significant difference in the levels of total IgA in sputum in the presence or absence of SARS-CoV-2 (**Figure 2E**). Even if there are differences in IgA expression by different EV subpopulations, this may not be reflected in the analysis of total proteins.

Clustering of individual EVs obtained from all samples was displayed in an tSNE plot in which 40 clusters were color-labeled (**Figure 3B**). After quantifying the percentage of each subpopulation, we found significant differences in 18 clusters (**Figure S3**).

EVs in cluster 2, which comprised 4.92% of all EVs in the nCOV group and only 0.55% of all EVs in the HC group (**Figures 5A**, **S3**), contained a large amount of protein SARS-CoV-2 N, suggesting that these EVs transport SARS-CoV-2 directly. Cluster 2 expressed high levels of EpCAM, CDH1, ITGB4, ITGA5, SNAI2, CD81, ITGB2, ZEB1, CD151, and MAdCAM-1. A previous study showed that CDH1 is required for HCV infection; indeed, silencing CDH1 significantly inhibited HCV infection of primary human hepatocytes at the postbinding entry step (22). Furthermore, ITGB2, ITGB3, and CD151 are involved in vesicle internalization and recycling to the cell membrane (23–25). ITGB3 plays a central role in intracellular communication through EVs (26). Meanwhile, CD151 plays a critical role in influenza A virus signaling (27), and ITGB4 participates in cell recognition through CD81 (21). The present results show that these molecules are highly expressed by cluster 2. Therefore, we boldly speculate that high expression of adhesion proteins such as EpCAM and CDH1 (**Figures 2F**, **S2**) by EVs may make individuals more susceptible to SARS-CoV-2 infection. These adhesion factors improve the recognition function of EVs; thus, these EVs are more likely to carry virus particles and be absorbed by recipient cells. Once these EVs are taken up by epithelial cells, cellular expression of CDH1 would be reduced due to virus-induced epithelial to mesenchymal transition (EMT) (28). Indeed, we also noted high expression of ZEB1 and Snai2. In addition, abnormal expression of integrins plays an important role in fibrosis formation (29, 30). Histological examination of biopsy samples obtained from COVID-19 patients revealed bilateral diffuse alveolar damage, with cellular fibromyxoid exudates (31), for which the processes mentioned above may be partly responsible.

In addition to cluster 2, clusters 3, 4, 6, 7, 9, 10, 12, 13, 14, 32, and 34 increased in the nCOV group, showing high expression of adhesion molecules (**Figures 5C**, S3). Adhesion molecules are involved in various important physiological functions and pathological processes, including leukocyte adhesion to vascular endothelial cells and lymphocyte homing during inflammation (20). These processes are controlled by integrin binding to endothelial and mucosal ligands (e.g., integrin α4β7 and MAdCAM-1) (32). After SARS-CoV-2 infection, EVs may trigger new multistep adhesion cascades, leading to inflammation. Which needs to be verified in follow-up experiments.

With respect to the EVs themselves, they are regulated by surface markers (CD9, CD63, CD81, and ALIX) (33), although different factors regulate different EV functions (34, 35). We found that EVs regulated by CD81 were more likely to bind to SARS-CoV-2 N protein (**Figure 6C**). In addition, clusters 2 (**Figure 5B**), 4 (**Figure 5C**), 6, 7, 12, and 34 (**Figure S3**) all showed upregulated protein expression after SARS-CoV-2 infection, and all expressed high levels of CD81. These results suggest that EVs regulated by CD81 are the most likely subpopulations to cause changes in the pulmonary microenvironment after SARS-CoV-2 infection.

Furthermore, HCV, which has been studied extensively, enters the host cell by interacting with a cascade of cellular factors, including CDH1, claudin-1 (CLDN1), and occludin (OCLN) (22). The virus is then taken up by recipient cells. These transmission processes may be similar to those used by SARS-CoV-2; indeed, EVs regulated by CD81 also express high amounts of CDH1. The difference is that EGFR is not required for the transmission of SARS-CoV-2, whereas it is required for transmission of HCV (**Figure 6A**). HCV uses a dynamic and multistep process to engage and enter host cells, in which EGFR is necessary for internalization (36).

Due to the special nature of SARS-CoV-2, we could not conduct general operations and experiments in ordinary environment. We used PBA technology to analyze the proteomics of individual exosomes, but the limitation of this technology is that we can only analyze 100 proteins designed in the panel (included typical EV biomarkers, biomarkers related to lung diseases, and a panel of cell adhesion molecules), which limits the analysis of all proteins to a certain extent. In addition, the experiment only took one time point and did not observe other time points or the prognosis of patients. According to the inspiration of this study, more other studies can be carried out. For example, drug interventions can target EVs that express CD81. This study has demonstrated that EVs are involved in the immune response of SARS-CoV-2, and that CD81-regulated EV subpopulation are the most likely to correlate with changes in the pulmonary microenvironment after SARS-CoV-2 infection. We can consider the development of nanomaterial vesicle-like antibody drugs that express CD81. Theoretically, these vesicles will be more fused with SARS-CoV-2 *in vivo* to achieve the effect of intervention.

In conclusion, we found that EVs (mostly regulated by CD81) can carry the SARS-CoV-2 N protein, and that expression of SARS-CoV N in EVs is associated strongly with that of inflammatory factors. These results demonstrate that EVs derived from the sputum of patients may participate in virus infection and immune responses. Taken together, the data presented herein may facilitate further study of COVID-19 and increase our understanding of disease pathogenesis.

## Data availability statement

The datasets presented in this study can be found in online repositories. The names of the repository/repositories and accession number(s) can be found below: https://doi.org/10.5061/dryad.qjq2bvqkf.

## Ethics statement

The studies involving human participants were reviewed and approved by the ethical standards of the Medical Ethical Council of the First Affiliated Hospital of Guangzhou Medical University. The patients/participants provided their written informed consent to participate in this study.

## Author contributions

PR, RS, DW, and JZ conceived the study; RS, YZ, GB, JS, PK, YiL, and AZ collected clinical specimens and performed the experiments; RS, YC, YZ, GB, PK, YuL, WL, JL, NC, JX, and DW analyzed the data; JZ, BL, and YZ contributed to critical revision of the manuscript; PR, RS, YC, and DW wrote the manuscript. All authors contributed to the article and approved the submitted version.
