# Supplementary material for: Proteomic profiling of single extracellular vesicles reveals colocalization of SARS-CoV-2 with a CD81/integrin-rich EV subpopulation in sputum from COVID-19 severe patients
Source: Front Immunol. 2023 May 12;14:1052141. doi: 10.3389/fimmu.2023.1052141 (PMC10214957; doi:10.3389/fimmu.2023.1052141)
Supplement: Supplementary file 1 [file DataSheet_1.pdf]

Supplementary information

Table 1. protein under investigation and DNA barcode on antibodies.

protein name	Conjugated DNA sequences
Caveolin-1	AAGCGTGTGATTCCTAGTAATGCTAGTGCGNNNNNNNNNTGCTATTATGATGTCTCAGGT
ITGA1	AAGCGTGTGATTCCTAGTAATGACCTAGCGNNNNNNNNNTGCTATTATGATGTCTCAGGT
ITGA2	AAGCGTGTGATTCCTAGTAATGTTAAGCCGNNNNNNNNNTGCTATTATGATGTCTCAGGT
ITGA5	AAGCGTGTGATTCCTAGTAATGGCCATACGNNNNNNNNNTGCTATTATGATGTCTCAGGT
ITGA6	AAGCGTGTGATTCCTAGTAATGTTCACACGNNNNNNNNNTGCTATTATGATGTCTCAGGT
ITGA9	AAGCGTGTGATTCCTAGTAATGGTCATTAGNNNNNNNNNTGCTATTATGATGTCTCAGGT
ITGAL	AAGCGTGTGATTCCTAGTAATGCTATATAGNNNNNNNNNTGCTATTATGATGTCTCAGGT
ITGAV	AAGCGTGTGATTCCTAGTAATGTACTCGAGNNNNNNNNNTGCTATTATGATGTCTCAGGT
ITGB1	AAGCGTGTGATTCCTAGTAATGCGTAAGAGNNNNNNNNNTGCTATTATGATGTCTCAGGT
ITGB2	AAGCGTGTGATTCCTAGTAATGTCATGCAGNNNNNNNNNTGCTATTATGATGTCTCAGGT
ITGB3	AAGCGTGTGATTCCTAGTAATGCATATAAGNNNNNNNNNTGCTATTATGATGTCTCAGGT
ITGB4	AAGCGTGTGATTCCTAGTAATGTATCCAAGNNNNNNNNNTGCTATTATGATGTCTCAGGT
ITGB5	AAGCGTGTGATTCCTAGTAATGACCGCTTCNNNNNNNNNTGCTATTATGATGTCTCAGGT
ITGB6	AAGCGTGTGATTCCTAGTAATGCGTAATTCNNNNNNNNNTGCTATTATGATGTCTCAGGT
ITGB7	AAGCGTGTGATTCCTAGTAATGTACCGGTCNNNNNNNNNTGCTATTATGATGTCTCAGGT
CD9	AAGCGTGTGATTCCTAGTAATGGCGAAGTCNNNNNNNNNTGCTATTATGATGTCTCAGGT
ITGAM	AAGCGTGTGATTCCTAGTAATGGTTGACTCNNNNNNNNNTGCTATTATGATGTCTCAGGT
CD24	AAGCGTGTGATTCCTAGTAATGCCTAGATCNNNNNNNNNTGCTATTATGATGTCTCAGGT
CD26	AAGCGTGTGATTCCTAGTAATGAAGCCATCNNNNNNNNNTGCTATTATGATGTCTCAGGT
CD26	AAGCGTGTGATTCCTAGTAATGACAATTGCNNNNNNNNNTGCTATTATGATGTCTCAGGT
CD36	AAGCGTGTGATTCCTAGTAATGTAAGATGCNNNNNNNNNTGCTATTATGATGTCTCAGGT
CD63	AAGCGTGTGATTCCTAGTAATGTTGACGGCNNNNNNNNNTGCTATTATGATGTCTCAGGT
CD73	AAGCGTGTGATTCCTAGTAATGAGACTCGCNNNNNNNNNTGCTATTATGATGTCTCAGGT
CD81	AAGCGTGTGATTCCTAGTAATGAGTAACGCNNNNNNNNNTGCTATTATGATGTCTCAGGT
CD90/Thy1	AAGCGTGTGATTCCTAGTAATGACCTGAGCNNNNNNNNNTGCTATTATGATGTCTCAGGT
LAMP2	AAGCGTGTGATTCCTAGTAATGTGTAGTCCNNNNNNNNNTGCTATTATGATGTCTCAGGT
ErbB2/Her2	AAGCGTGTGATTCCTAGTAATGTGACTGCCNNNNNNNNNTGCTATTATGATGTCTCAGGT
TROP-2	AAGCGTGTGATTCCTAGTAATGCACTAGCCNNNNNNNNNTGCTATTATGATGTCTCAGGT
EpCAM	AAGCGTGTGATTCCTAGTAATGTACGCACCNNNNNNNNNTGCTATTATGATGTCTCAGGT
Glypican 1	AAGCGTGTGATTCCTAGTAATGAGTATTACNNNNNNNNNTGCTATTATGATGTCTCAGGT
EpCAM	AAGCGTGTGATTCCTAGTAATGATGACTACNNNNNNNNNTGCTATTATGATGTCTCAGGT
Cadhherin	AAGCGTGTGATTCCTAGTAATGACGTGGACNNNNNNNNNTGCTATTATGATGTCTCAGGT
IL-6	AAGCGTGTGATTCCTAGTAATGCCTTAGACNNNNNNNNNTGCTATTATGATGTCTCAGGT
CLEC-2	AAGCGTGTGATTCCTAGTAATGATTAGCACNNNNNNNNNTGCTATTATGATGTCTCAGGT
CLEC-2A	AAGCGTGTGATTCCTAGTAATGCGTTGAACNNNNNNNNNTGCTATTATGATGTCTCAGGT
EGF R	AAGCGTGTGATTCCTAGTAATGCTGCCTTANNNNNNNNNTGCTATTATGATGTCTCAGGT
cadherin-17	AAGCGTGTGATTCCTAGTAATGCCATTGTTNNNNNNNNNTGCTATTATGATGTCTCAGGT
ITGA4B7	AAGCGTGTGATTCCTAGTAATGACTCGGTTNNNNNNNNNTGCTATTATGATGTCTCAGGT
ESAM	AAGCGTGTGATTCCTAGTAATGAATGCGTTNNNNNNNNNTGCTATTATGATGTCTCAGGT
CD20	AAGCGTGTGATTCCTAGTAATGATGCAGTTNNNNNNNNNTGCTATTATGATGTCTCAGGT
CLDN4	AAGCGTGTGATTCCTAGTAATGTGAATCTTNNNNNNNNNTGCTATTATGATGTCTCAGGT

ULBP-3	AAGCGTGTGATTCCTAGTAATGGAGTCCTTNNNNNNNNNTGCTATTATGATGTCTCAGGT
ADAM10	AAGCGTGTGATTCCTAGTAATGGCGATATTNNNNNNNNNTGCTATTATGATGTCTCAGGT
CDH2	AAGCGTGTGATTCCTAGTAATGCAGCGATTNNNNNNNNNTGCTATTATGATGTCTCAGGT
AMGO1	AAGCGTGTGATTCCTAGTAATGTACCTTGTNNNNNNNNNTGCTATTATGATGTCTCAGGT
CTLA-4	AAGCGTGTGATTCCTAGTAATGCACGGTGTNNNNNNNNNTGCTATTATGATGTCTCAGGT
IgA	AAGCGTGTGATTCCTAGTAATGGACACTGTNNNNNNNNNTGCTATTATGATGTCTCAGGT
ITGB8	AAGCGTGTGATTCCTAGTAATGACCTTGGTNNNNNNNNNTGCTATTATGATGTCTCAGGT
MAdCAM-1	AAGCGTGTGATTCCTAGTAATGAACCAGGTNNNNNNNNNTGCTATTATGATGTCTCAGGT
CD44	AAGCGTGTGATTCCTAGTAATGCCATGCGTNNNNNNNNNTGCTATTATGATGTCTCAGGT
ITGA8	AAGCGTGTGATTCCTAGTAATGAGGACCGTNNNNNNNNNTGCTATTATGATGTCTCAGGT
Siglec-5	AAGCGTGTGATTCCTAGTAATGACGCTAGTNNNNNNNNNTGCTATTATGATGTCTCAGGT
DSG3	AAGCGTGTGATTCCTAGTAATGTTGGCAGTNNNNNNNNNTGCTATTATGATGTCTCAGGT
ITGAX	AAGCGTGTGATTCCTAGTAATGGAGGTTCTNNNNNNNNNTGCTATTATGATGTCTCAGGT
ITGA11	AAGCGTGTGATTCCTAGTAATGGATTCTCTNNNNNNNNNTGCTATTATGATGTCTCAGGT
CEACAM-8	AAGCGTGTGATTCCTAGTAATGGACCATCTNNNNNNNNNTGCTATTATGATGTCTCAGGT
FOLH1	AAGCGTGTGATTCCTAGTAATGACATGGCTNNNNNNNNNTGCTATTATGATGTCTCAGGT
ITGB4	AAGCGTGTGATTCCTAGTAATGATTGAGCTNNNNNNNNNTGCTATTATGATGTCTCAGGT
CEACAM-6	AAGCGTGTGATTCCTAGTAATGGGTATCCTNNNNNNNNNTGCTATTATGATGTCTCAGGT
PAR2	AAGCGTGTGATTCCTAGTAATGAGTCACCTNNNNNNNNNTGCTATTATGATGTCTCAGGT
LYN	AAGCGTGTGATTCCTAGTAATGAATGGACTNNNNNNNNNTGCTATTATGATGTCTCAGGT
ITGA5	AAGCGTGTGATTCCTAGTAATGGTAGCACTNNNNNNNNNTGCTATTATGATGTCTCAGGT
CD151	AAGCGTGTGATTCCTAGTAATGAGCGGTATNNNNNNNNNTGCTATTATGATGTCTCAGGT
nectin1	AAGCGTGTGATTCCTAGTAATGGCTGCTATNNNNNNNNNTGCTATTATGATGTCTCAGGT
ITGA6	AAGCGTGTGATTCCTAGTAATGCCTCTGATNNNNNNNNNTGCTATTATGATGTCTCAGGT
ADSF	AAGCGTGTGATTCCTAGTAATGATATCGATNNNNNNNNNTGCTATTATGATGTCTCAGGT
ITGA4	AAGCGTGTGATTCCTAGTAATGGCGCAGATNNNNNNNNNTGCTATTATGATGTCTCAGGT
ITGB1	AAGCGTGTGATTCCTAGTAATGCTACGCATNNNNNNNNNTGCTATTATGATGTCTCAGGT
JAM-B	AAGCGTGTGATTCCTAGTAATGCACGTAATNNNNNNNNNTGCTATTATGATGTCTCAGGT
ITGA3	AAGCGTGTGATTCCTAGTAATGACCTGTTGNNNNNNNNNTGCTATTATGATGTCTCAGGT
DSG4	AAGCGTGTGATTCCTAGTAATGTTACCTTGNNNNNNNNNTGCTATTATGATGTCTCAGGT
LAMP2	AAGCGTGTGATTCCTAGTAATGCTTCATTGNNNNNNNNNTGCTATTATGATGTCTCAGGT
DSG1	AAGCGTGTGATTCCTAGTAATGAGACTGTGNNNNNNNNNTGCTATTATGATGTCTCAGGT
DSG2	AAGCGTGTGATTCCTAGTAATGCGAACGTGNNNNNNNNNTGCTATTATGATGTCTCAGGT
IgM	AAGCGTGTGATTCCTAGTAATGGATGTCTGNNNNNNNNNTGCTATTATGATGTCTCAGGT
PCDH15	AAGCGTGTGATTCCTAGTAATGAGAGCCTGNNNNNNNNNTGCTATTATGATGTCTCAGGT
HLAA	AAGCGTGTGATTCCTAGTAATGATCGTATGNNNNNNNNNTGCTATTATGATGTCTCAGGT
CLDN19	AAGCGTGTGATTCCTAGTAATGCAGTCATGNNNNNNNNNTGCTATTATGATGTCTCAGGT
CLP24	AAGCGTGTGATTCCTAGTAATGCAGGTTGGNNNNNNNNNTGCTATTATGATGTCTCAGGT
CEACAM-4	AAGCGTGTGATTCCTAGTAATGCATACTGGNNNNNNNNNTGCTATTATGATGTCTCAGGT
L1CAM/CD171	AAGCGTGTGATTCCTAGTAATGCGTTACGGNNNNNNNNNTGCTATTATGATGTCTCAGGT
Siglec-8	AAGCGTGTGATTCCTAGTAATGTTATCAGGNNNNNNNNNTGCTATTATGATGTCTCAGGT
ITGA2	AAGCGTGTGATTCCTAGTAATGGAACTTCGNNNNNNNNNTGCTATTATGATGTCTCAGGT
uPA	AAGCGTGTGATTCCTAGTAATGACAGCTCGNNNNNNNNNTGCTATTATGATGTCTCAGGT
SARS N	AAGCGTGTGATTCCTAGTAATGGGTGTCGANNNNNNNNNTGCTATTATGATGTCTCAGGT
F	AAGCGTGTGATTCCTAGTAATGTCTTACGANNNNNNNNNTGCTATTATGATGTCTCAGGT

P	AAGCGTGTGATTCCTAGTAATGTAGTCAGANNNNNNNNNTGCTATTATGATGTCTCAGGT
ACE2	AAGCGTGTGATTCCTAGTAATGCATGGTCANNNNNNNNNTGCTATTATGATGTCTCAGGT
Alix	AAGCGTGTGATTCCTAGTAATGCGGCTGTANNNNNNNNNTGCTATTATGATGTCTCAGGT
tsg101	AAGCGTGTGATTCCTAGTAATGACAGCGTANNNNNNNNNTGCTATTATGATGTCTCAGGT
snail	AAGCGTGTGATTCCTAGTAATGCCGGTCTANNNNNNNNNTGCTATTATGATGTCTCAGGT
FN	AAGCGTGTGATTCCTAGTAATGCGAGGCTANNNNNNNNNTGCTATTATGATGTCTCAGGT
slug	AAGCGTGTGATTCCTAGTAATGTCACGATANNNNNNNNNTGCTATTATGATGTCTCAGGT
Eca	AAGCGTGTGATTCCTAGTAATGAAGCTTGANNNNNNNNNTGCTATTATGATGTCTCAGGT
ZEB-1	AAGCGTGTGATTCCTAGTAATGCCGGATGANNNNNNNNNTGCTATTATGATGTCTCAGGT
TGFβ	AAGCGTGTGATTCCTAGTAATGGTAGCGGANNNNNNNNNTGCTATTATGATGTCTCAGGT
ZO-1	AAGCGTGTGATTCCTAGTAATGTGGAGCGANNNNNNNNNTGCTATTATGATGTCTCAGGT
vim	AAGCGTGTGATTCCTAGTAATGCCATTAGANNNNNNNNNTGCTATTATGATGTCTCAGGT
wnt5α	AAGCGTGTGATTCCTAGTAATGGCTGCAGANNNNNNNNNTGCTATTATGATGTCTCAGGT
CC10	AAGCGTGTGATTCCTAGTAATGGCTCGTCANNNNNNNNNTGCTATTATGATGTCTCAGGT

Table 2. Gene name and uniprot id

	**gene name**	**uniprot id**	**Entrez Gene ID**
1	CDH17	Q12864	1015
2	ITGA4B7		3676
3	ESAM	Q96AP7	90952
4	CD20	P11836	931
5	CLDN4	O14493	1364
6	ULBP3	Q9BZM4	79465
7	ADAM10	O14672	102
8	CDH2	P19022	1000
9	AMIGO1	Q86WK6	57463
10	CTLA4	P16410	1493
11	IgA		
12	ITGB8	P26012	3696
13	MADCAM1	Q13477	8174
14	CD44	P16070	960
15	ITGA8	P53708	8516
16	SIGLEC5	O15389	8778
17	DSG3	P32926	1830
18	ITGAX	P20702	3687
19	ITGA11	Q9UKX5	22801
20	CEACAM8	P31997	1088
21	FOLH1	Q04609	2346
22	ITGB4	P16144	3691
23	CEACAM6	P40199	4680
24	PAR2	P55085	2150

25	LYN	P07948	4067
26	ITGA5	P08648	3678
27	CD151	P48509	977
28	NECTIN1	Q15223	5818
29	ITGA6	P23229	3655
30	RETN	Q9HD89	56729
31	ITGA4	P13612	3676
32	ITGB1	P05556	3688
33	JAM2	P57087	58494
34	ITGA3	P26006	3675
35	DSG4	Q86SJ6	147409
36	LAMP2	P13473	3920
37	DSG1	Q02413	1828
38	DSG2	Q14126	1829
39	IgM		
40	PCDH15	Q96QU1	65217
41	HLA-A	P04439	3105
42	CLDN19	Q8N6F1	149461
43	TMEM204	Q9BSN7	79652
44	CEACAM4	O75871	1089
45	L1CAM	P32004	3897
46	SIGLEC8	Q9NYZ4	27181
47	ITGA2	M3YSD9	3673
48	PLAU	G9KH01	5328
49	CAV1	Q03135	857
50	ITGA1	P56199	3672
51	ITGA2	P17301	3673
52	ITGA5	P08648	3678
53	ITGA6	P23229	3655
54	ITGA9	Q13797	3680
55	ITGAL	P20701	3683
56	ITGAV	P06756	3685
57	ITGB1	P05556	3688
58	ITGB2	P05107	3689
59	ITGB3	P05106	3690
60	ITGB4	P16144	3691
61	ITGB5	P18084	3693
62	ITGB6	P18564	3694
63	ITGB7	P26010	3695
64	CD9	P21926	928

65	ITGAM	P11215	3684
66	CD24	P25063	100133941
67	CD26	P27487	1803
68	CD26	P27487	1803
69	CD36	P16671	948
70	CD63	P08962	967
71	NT5E	P21589	4907
72	CD81	P60033	975
73	Thy1	P04216	7070
74	LAMP2	P13473	3920
75	ERBB2	P04626	2064
76	TACSTD2	P09758	4070
77	EPCAM	P16422	4072
78	GPC1	P35052	2817
79	EPCAM	P16422	4072
80	CDH15	P55291	1013
81	IL6	P05231	3569
82	CLEC1B	Q9P126	51266
83	CLEC2A	Q6UVW9	387836
84	EGFR	P00533	1956
85	ALIX	Q8WUM4	10015
86	TSG101	Q99816	7251
87	WNT5A	P41221	7474
88	ZO1	Q07157	7082
89	FN	P02751	2335
90	VIM	P08670	7431
91	CDH1	A0A0U2ZQU7	999
92	SNAI1	O95863	6615
93	ZEB1	P37275	6935
94	TGFBR1	P36897	7046
95	SNAI2	O43623	6591
96	SCGB1A1	P11684	7356
97	PHB2	Q99623	11331
98	FAP	Q12884	2191
99	ACE2	Q9BYF1	59272
100	SARS-CoV-2 N		

For each single or pair of **FastQ** files, AfterQC filters out bad reads, detects and eliminates sequencer's bubble effects, trims reads at front and tail, detects the sequencing errors and corrects part of them, and finally outputs clean data
