# Supplementary figures and images for: Proteomic profiling of single extracellular vesicles reveals colocalization of SARS-CoV-2 with a CD81/integrin-rich EV subpopulation in sputum from COVID-19 severe patients
Source: Front Immunol. 2023 May 12;14:1052141. doi: 10.3389/fimmu.2023.1052141 (PMC10214957; doi:10.3389/fimmu.2023.1052141)

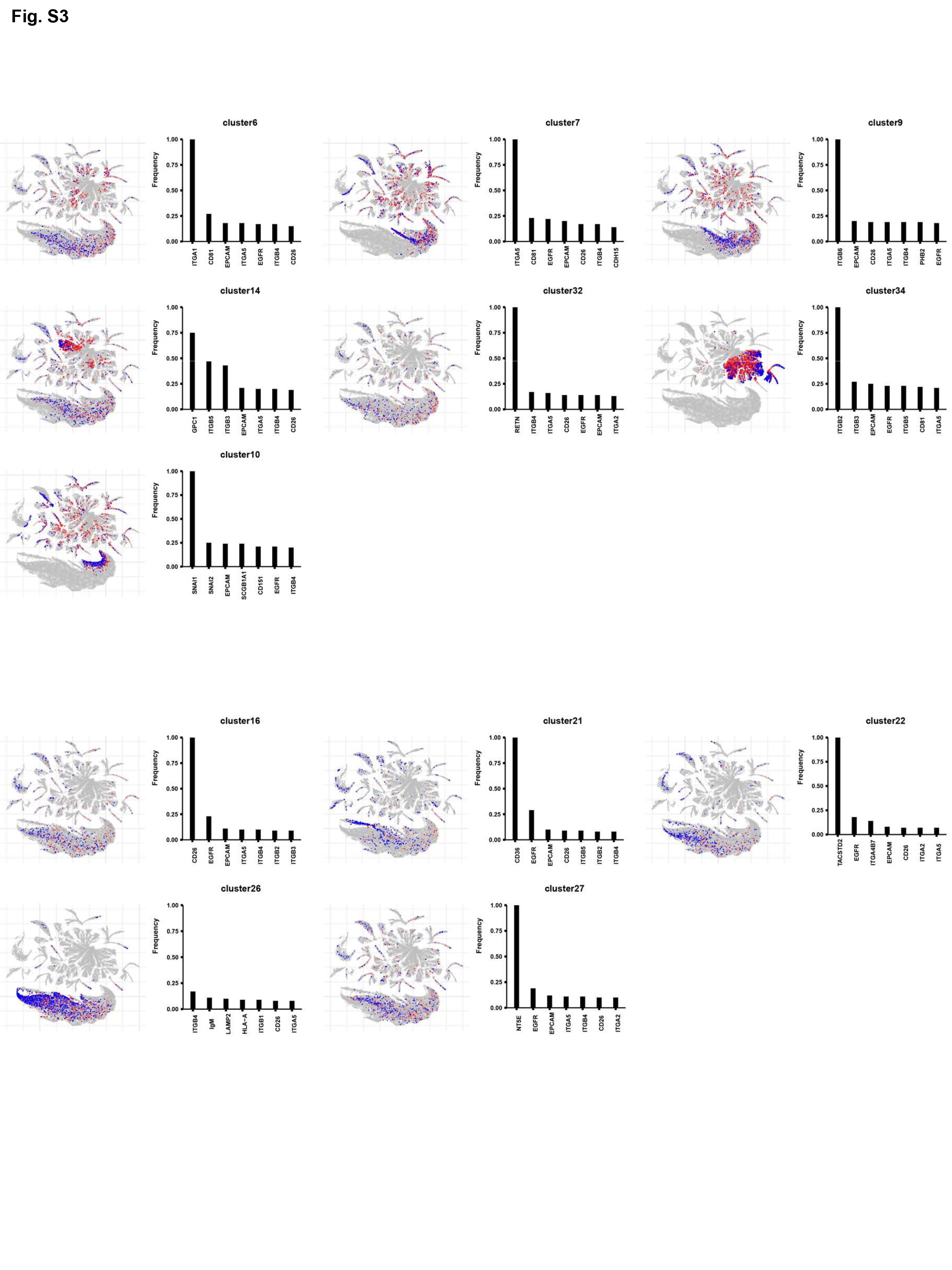

Supplement: Supplementary file 2 [file DataSheet_2.zip › Figure S3.JPEG]

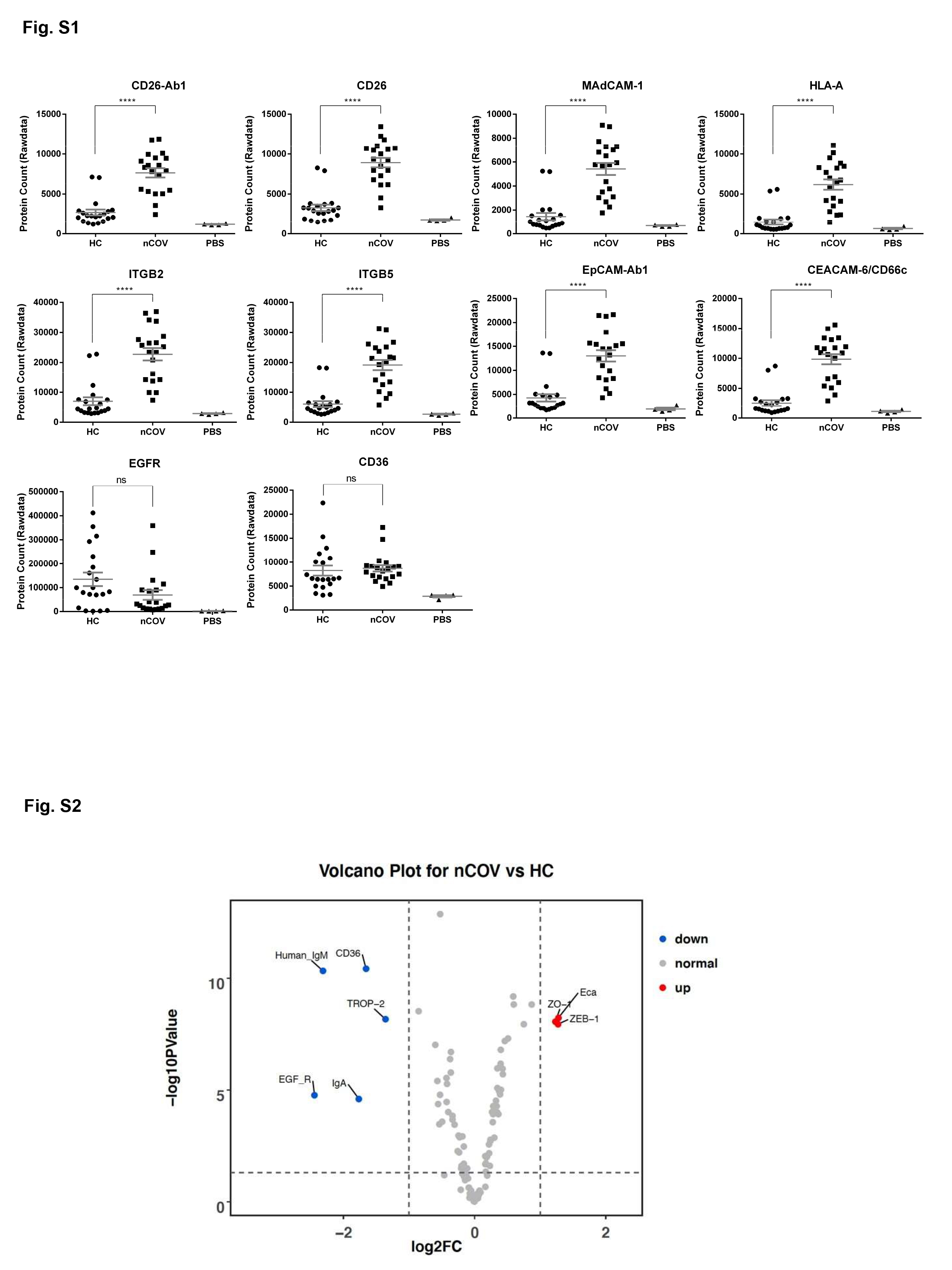

Supplement: Supplementary file 2 [file DataSheet_2.zip › Figures S1 and S2.JPEG]
